# Investigating the temporal trends of diabetes disease burden in China during 1990-2019 from a global perspective

**DOI:** 10.3389/fendo.2024.1324318

**Published:** 2024-05-10

**Authors:** Jinli Liu, Mingwang Shen, Guihua Zhuang, Lei Zhang

**Affiliations:** ^1^ Department of Endocrinology, the First Affiliated Hospital of Xi'an Jiaotong University, Xi’an, Shaanxi, China; ^2^ China-Australia Joint Research Center for Infectious Diseases, School of Public Health, Xi'an Jiaotong University Health Science Center, Xi’an, Shaanxi, China; ^3^ Key Laboratory of Environment and Genes Related to Diseases (Xi'an Jiaotong University), Ministry of Education, Xi’an, China; ^4^ Melbourne Sexual Health Centre, Alfred Health, Melbourne, VIC, Australia; ^5^ Central Clinical School, Faculty of Medicine, Monash University, Melbourne, VIC, Australia

**Keywords:** diabetes, disease burden, estimated annual percentage changes, decomposition analysis, China

## Abstract

**Introduction:**

Diabetes poses a global public health challenge and our understanding of its temporal evolution in China relative to the rest of the world is limited. Our study aims to comprehensively examine the temporal trend of diabetes DALYs in China from a global perspective.

**Methods:**

We analyzed data on diabetes incidence, prevalence, and mortality for individuals aged ≥20 years in China and globally from the Global Burden of Disease (GBD) 2019 study. We assessed trends in age-standardized incidence rate (ASIR) and age-standardized mortality rate (ASMR) of diabetes in China and globally by estimating annual percentage changes (EAPCs). We employed decomposition analysis to reveal factors driving the trend of diabetes DALYs in China.

**Results:**

During 1990-2019, the number of diabetes patients increased by 160% from 35.14 million to 91.70 million in China. The ASIR of diabetes increased from 249 per 100,000 to 329 per 100,000 in China, which was lower than the global rate (419 per 100,000 in 2019). The EAPC of diabetes incidence was also lower in China compared to the global rate (1.02% vs. 1.57%). Consistently, the age-standardized prevalence rate of diabetes increased from 4788 per 100,000 to 8170 per 100,000 during 1990-2019 in China, which remained lower than the corresponding global rate (8827 per 100,000 in 2019). Further, the ASMR of diabetes increased from 9 per 100,000 to 15 per 100,000 during 1990-2019 in China, which was lower than the corresponding global rate (30 per 100,000 in 2019). However, EAPC of diabetes mortality in China was much higher than the global level (1.75% vs. 1.07%). Globally, the rising diabetes DALYs was predominantly attributed to population growth (55.2%) and epidemiologic changes (24.6%). In comparison, population growth (48.9%) also played an important role in the increasing diabetes DALYs in China, but aging (43.7%) was second major contributor.

**Conclusion:**

Our findings show that diabetes DALYs in China followed a global increasing trend during 1990-2019. Notably, aging has a very substantial contribution to the increase in diabetes DALYs in China in addition to population growth.

## Introduction

Diabetes has emerged as a critical global health challenge in the 21st century. Worldwide, the number of diabetes cases surged by 102.9%, from 11.3 million in 1990 to 22.9 million in 2017 (1). In China, the incidence of diabetes rose from 177 per 100,000 in 1990 to 199 per 100,000 in 2017 (1). Accumulating evidence demonstrates a global escalation in diabetes prevalence, which is attributable to a complex interplay of multiple factors (2). As the country with the largest diabetic population, China witnessed a rise in prevalence from 2.6% in 2002 to 10.9% in 2013 then to 12.4% in 2018 (3, 4), Globally, diabetes-related deaths among adults amounted to 3.96 million deaths in 2010 (5) and this number increased to 4.20 million in 2019 (6). Diabetes contributes a large spectrum complications, particularly diabetic nephropathy and cardiovascular conditions. The comorbidities of diabetes can cause further disease and economic burdens to the society, in addition to the burdens caused by diabetes (7). The global burden of diabetes in DALYs amounted to 79.2 million in 2021, with China accounting for 15% of cases (2).

The severe disease burden of diabetes was expected to cause a heavy economic burden. Liu et al. predicted that the total costs associated with diabetes would increase from $250.2 billion to $460.4 billion in China during 2020-2030, corresponding to an annual growth rate of 6.32% (8). As a multitude of factors contribute to the onset of diabetes, and without intervention, the burden of diabetes will continue to mount. In response, the Chinese State Council introduced the “Health China 2030” strategy in 2016, aiming to bolster chronic disease prevention and control initiatives and alleviate disease burden across China.

To date, a comprehensive understanding of the historical development of diabetes in China in a global context remains lacking. Acquiring insights into the evolving trends of diabetes DALYs in China from a global perspective is crucial for guiding prevention, treatment, and management strategies. In this study, we aimed to assess the temporal patterns of diabetes DALYs in China over a 30-year span (1990–2019) from a global perspective and elucidate the evolving dynamics of diabetes disease burden in the country. We employed geospatial information visualization techniques and natural log-linear regression models in our analysis.

## Materials and methods

### Data source and cleaning

The Global Burden of Disease Study 2019 (GBD 2019) provided estimates for various epidemiological indicators, including age-standardized incidence, prevalence, mortality, and disability-adjusted life-years (DALYs) for 23 age groups, males, females, and both sexes combined across 203 countries and territories. The couwere organized into 21 regions and seven super-regions (9). We extracted country-level data on incidence, prevalence, mortality and DALYs indicators (age-standardized rates and cases) for diabetes (including both type 1 and type 2 diabetes) between 1990 and 2019 using the Global Health Data Exchange (GHDx) query tool (https://ghdx.healthdata.org/gbd-2019). The definition and classification of diabetes were based on GBD research criteria (9). The definitions and diagnostic criteria for overall diabetes were presented in the appendix section. Data were available from 204 countries and territories, which were categorized into 5 regions based on sociodemographic index (SDI) (9).

### Trend analysis-estimated annual percentage change

The estimated annual percentage change (EAPC) (1, 10) is commonly used as a summary measure of the age-standardized incidence and mortality (ASIR and ASMR) trend over a specified period. It is determined by fitting a regression line to the natural logarithm of the ASIR and ASMR: y = α+βx+ϵ, where y represents the natural logarithm of age-standardized rates, x denotes the calendar year, and β indicates the positive or negative changing trends. The EAPC was calculated as 100×[exp(β)–1], and its 95% confidence interval (CI) can also be derived from a linear regression model. The calendar year was utilized as a continuous predictor variable. EAPC was employed to assess the annual growth rate of diabetes ASIR and ASMR. In this study, the ASIR or ASMR was regarded as ‘increasing’ if both their EAPC and the lower limit of its 95% CI were >0%. Conversely, the ASIR or ASMR was deemed to be ‘decreasing’ if both their EAPC and the upper limit of its 95% CI were <0%; otherwise, the ASIR or ASMR was considered ‘uncertain’ over time. We calculated and ranked EAPCs for diabetes ASIR and ASMR across 203 countries (**Supplementary Table S1**).

### Decomposition analysis

We conducted decomposition analysis to carefully examine the causal determinants and their individual impacts on the increase of diabetes DALYs from 1990 to 2019. These determinants included the effects of population growth, aging, and epidemiologic changes. Detailed methodologies were outlined in the accompanying appendices.

All statistical analyses were performed using R v3.5.1 (https://www.r-project.org/). A p-value of less than 0.05 was considered statistically significant.

## Results

### Temporal trends of diabetes ASIR in China and globally

**Figure 1:**
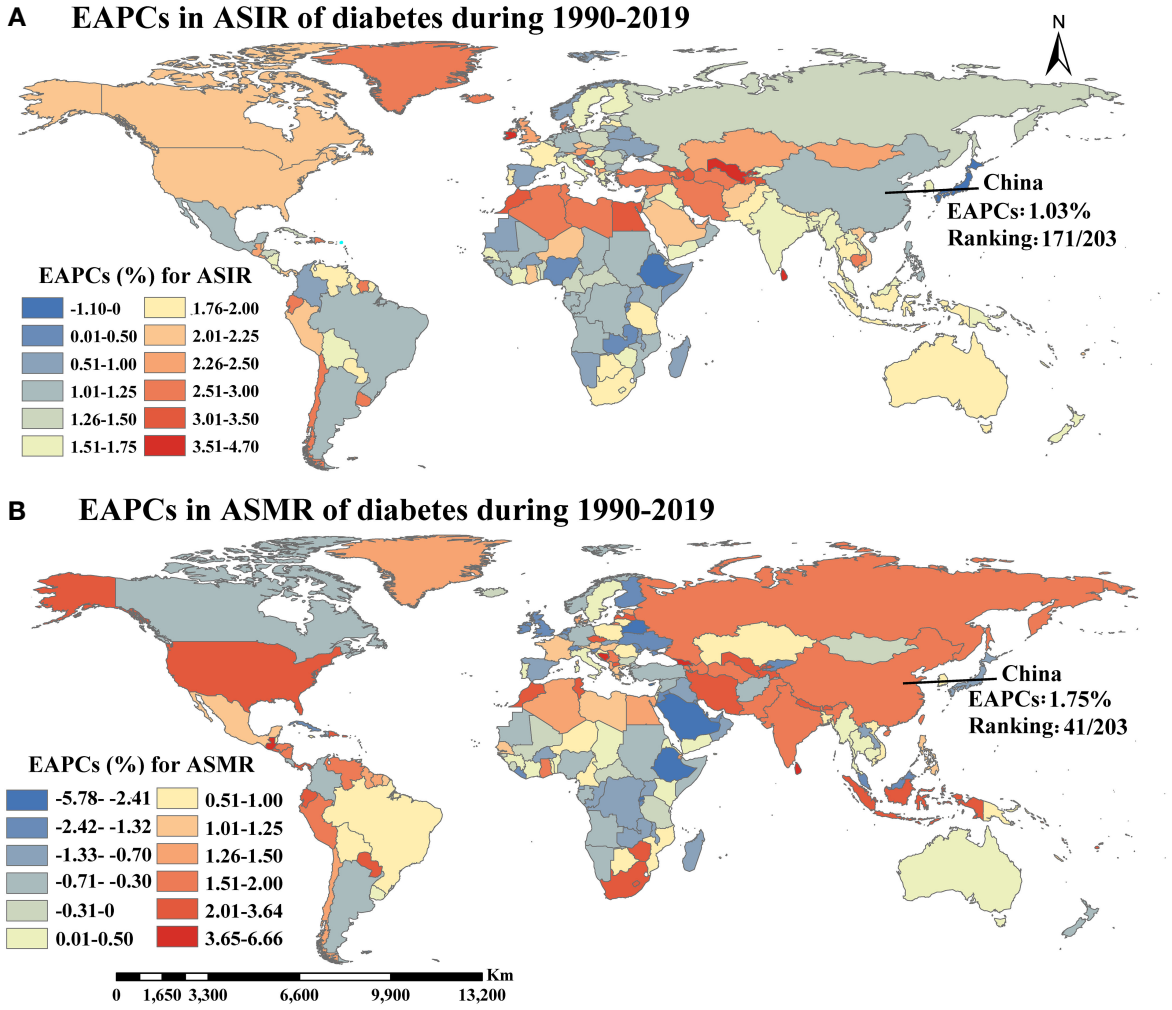
The temporal trends of ASIR **(A)** and ASMR **(B)** of diabetes during 1990-2019 (EAPCs, estimated annual percentage changes; ASIR, Age-standardized incidence rate; ASMR, Age-standardized mortality rate).

The temporal trend analysis of diabetes incidence data revealed that the ASIR of diabetes in the Chinese population exhibited an increasing trend until 2006, followed by a stabilized period up to 2015 then a significant decline from 2015 to 2017 (**Figure S1A**). The ASIR of diabetes increased from 249 (227–276) per 100,000 in 1990 to 329 (303–361) per 100,000 in 2019 among Chinese individuals aged ≥20 years (**Table 1**, **Supplementary Table S1**, **Supplementary Figure S2**). China’s ASIR ranking rose from 116th to 152nd (of 203 countries worldwide, ranked from highest to lowest ASIR) during 1990–2019. The EAPC of ASIR in China was 1.02% (95% CI, 0.79%–1.26%) during the same period, with a rank of 171st (**Figure 1A**, **Supplementary Table S1**).

**Table 1:** The disease burden of incidence, prevalence and mortality of diabetes in adults ≥20 years and their EAPC during 1990–2019 in China and Globally.

Characteristics	1990			2019			1990-2019
	CasesNo. million (95% UI)	Rate per 100,000 No.(95% UI)		Cases No. million (95% UI)	Rate per 100,000 No. (95% UI)		EAPCNo.(95% UI)
**China**							
ASIR	1.83(1.66–2.02)	249(227–276)		3.69(3.40–4.05)	329(303–361)		1.02 (0.79–1.26)
ASPR	35.14(31.64–39.06)	4788 (4311–5323)		91.70(83.99–100.23)	8170(7483–8930)		–
ASMR	0.07(0.06–0.08)	9 (8–11)		0.17(0.15–0.20)	15 (13–18)		1.75 (1.54–2.00)
**Global**							
ASIR	8.26(7.61–8.96)	269(247–291)		21.46(19.81–23.24)	416(384–451)		1.57 (1.53–1.65)
ASPR	156.01(143.35–169.60)	5071(4660–5513)		455.30(418.88–493.80)	8827(8121–9573)		–
ASMR	0.65(0.62–0.68)	21(20–22)		1.54(1.44–1.64)	30(28–32)		1.07 (1.01–1.15)

ASIR, Age-standardized incidence rate; ASPR, Age-standardized prevalence rate; ASMR, Age-standardized mortality rate.

**Figure 2:**
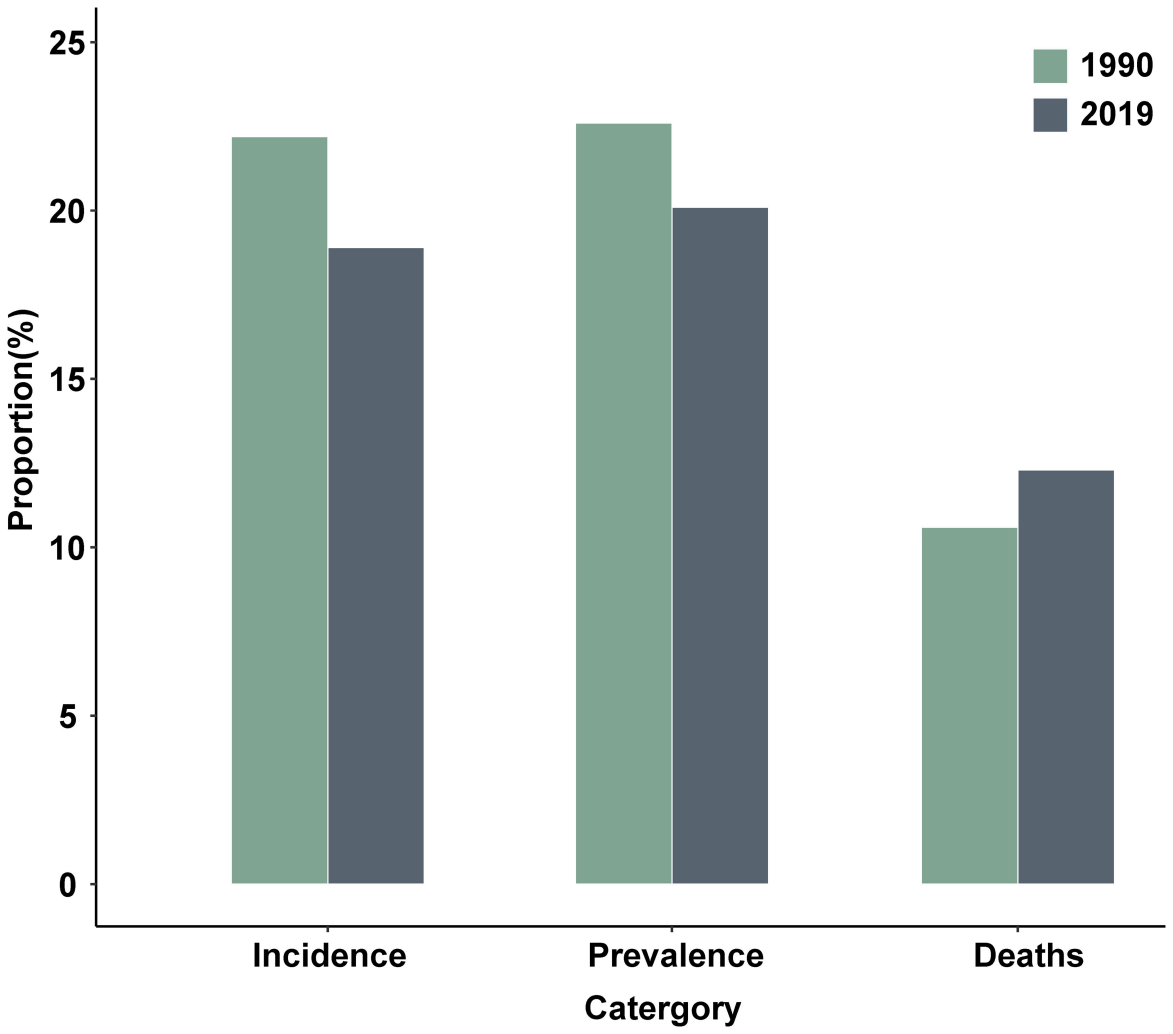
The number of diabetes new cases, patients and deaths in China as percentages of the corresponding indicators in the world (adjusting for the impact of the China’s population proportion).

Globally, the ASIR of diabetes in individuals aged ≥20 years increased from 269(247–291) per 100,000 in 1990 to 416(384–451) per 100,000 in 2019, with an EAPC of 1.57% (1.53%–1.65%) (**Table 1**, **Supplementary Table S1**). The number of new diabetes cases among individuals aged ≥20 years rose from 8.26 million in 1990 to 21.46 million in 2019 worldwide. After adjusting for China’s population proportion, the proportion of new cases in China relative to the global new cases decreased from 22.2% in 1990 to 18.9% in 2019 (**Figure 2**).

### Prevalence burden of diabetes in China and globally

The ASPR of diabetes among individuals ≥20 years old increased from 4788 (4311–5323) per 100,000 in 1990 (ranking 102nd) to 8170 (7483–8930) per 100,000 in 2019 (ranking 120th) in China, representing a 70.6% increase (**Table 1**, **Supplementary Table S1**, **Supplementary Figure S3**). The global ASPR of diabetes in individuals aged ≥20 years increased from 5071(4660–5513) per 100,000 in 1990 to 8827(8121–9573) per 100,000 in 2019, marking a 74.0% increase (**Table 1**). The number of individuals aged ≥20 years with diabetes in China increased from 35.14 million in 1990 to 91.70 million in 2019, the highest in the world for both years (**Supplementary Figure S4**). Globally, the number of individuals aged ≥20 years with diabetes increased from 156.01 million in 1990 to 455.30 million in 2019. After adjusting for the impact of the Chinese population proportion, the proportion of diabetes patients in China relative to the global total diabetes patients decreased from 22.6% in 1990 to 20.1% in 2019 (**Figure 2**).

### Temporal trends of diabetes ASMR in China and globally

In China, the ASMR of diabetes increased from 9(8–11) per 100,000 in 1990 to 15 (13–18) per 100,000 in 2019, with the world ranking declining from 192nd to 177th (**Table 1**, **Supplementary Table S1**, **Supplementary Figure S5**). The EAPC of diabetes ASMR in China was 1.75% (1.54%–2.00%) during 1990–2019, ranking 41st out of 203 countries worldwide (**Figure 1B**, **Supplementary Table S1**). A similar increasing trend of global diabetes ASMR (from 21(20–22) per 100,000 in 1990 to 30 (28–32) per 100,000 in 2019) was also observed over the same period, with an EAPC of 1.07% (1.01%–1.15%) (**Table 1**, **Supplementary Table S1**). Although the diabetes ASMR in China was considerably lower than the global rate, the EAPC of ASMR in China was higher. Approximately 0.07 million and 0.17 million individuals aged ≥20 years in China died from diabetes in 1990 and 2019, respectively, accounting for 10.6% and 12.3% of all diabetes deaths worldwide then (**Figure 2**).

### Drivers of diabetes epidemiology: population growth, aging, and epidemiologic changes

**Figure 3:**
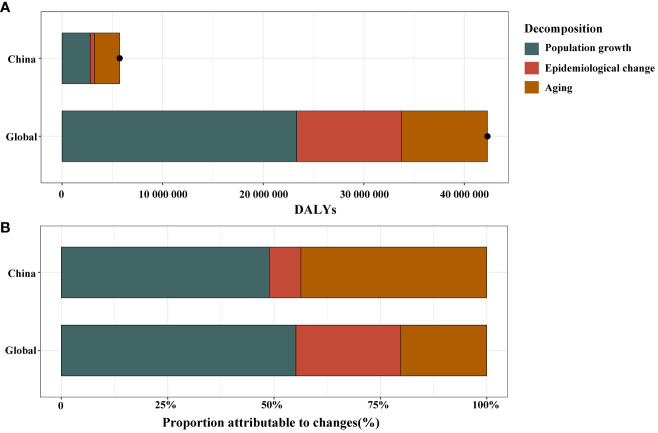
Changes in diabetes disability adjusted life years (DALYs) according to population-level determinants of population growth, aging, and epidemiological change from 1990 to 2019 in China and globally **(A)**. The black dot represents the overall value of change contributed by all 3 components. Proportion of the population-level determinants attributed to changes in diabetes DALYs from 1990 to 2019 in China and globally **(B)**.

Overall, diabetes DALYs witnessed a substantial increase in China and globally (**Figure 3**, **Supplementary Table S2**). Over the last thirty years, aging, population growth, and epidemiologic changes accounted for 2.5 million, 2.8 million, and 0.7 million DALYs in diabetes DALYs in China, respectively. Globally, these factors contributed 8.6 million, 23.3 million, and 10.4 million DALYs to the diabetes DALYs (**Figure 3A**, **Supplementary Table S2**). Population growth significantly drove changes of diabetes DALYs in China and globally, contributing 48.9% in China and 55.2% globally to the overall difference. The contribution of aging on diabetes DALYs was significantly greater in China (43.7%) compared to the global average (20.3%) (**Figure 3B**, **Supplementary Table S2**).

### The influential factors for EAPC

**Figure 4:**
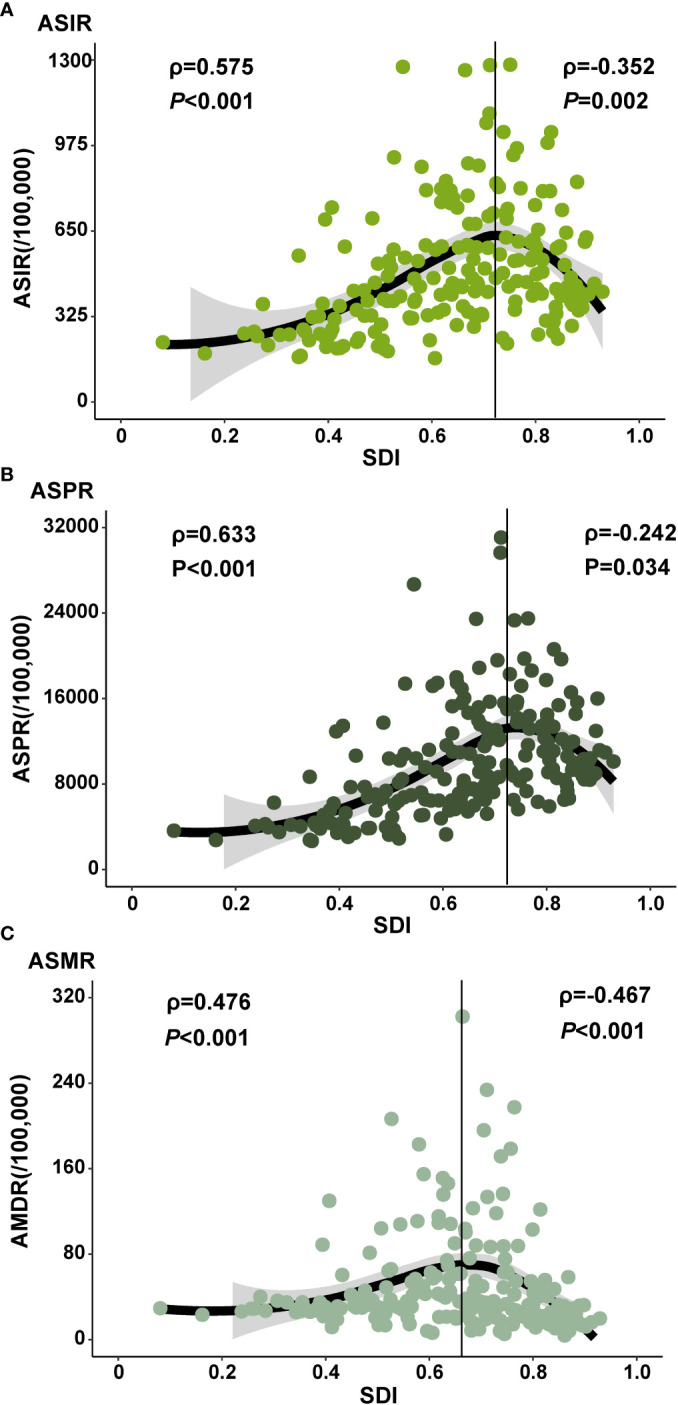
The ASIR, ASPR and ASMR of diabetes at national level. The correlation between sociodemographic index (SDI) in 2019 and ASIR **(A)** and ASPR **(B)** and ASMR **(C)** of diabetes in 2019. The ρ indices and P values presented in **(A-C)** were derived from Spearman correlation analysis. (ASIR: age-standardized incidence rate; ASPR: age-standardized prevalence rate; ASMR: age-standardized mortality rate).

As depicted in **Figure 4**, a significant association was observed between SDI and ASIR, ASPR, and ASMR in 2019, respectively. Interestingly, in countries with an SDI below 0.72, ASIR and SDI exhibited a positive correlation (ρ=0.575, *P*< 0.001). Conversely, in countries with a SDI surpassed 0.72, ASIR and SDI showed a negative correlation (ρ=-0.352, *P*=0.002). The relationship between ASPR and SDI followed a similar pattern. A notable positive association was detected between SDI and ASMR (ρ=0.476, *P*< 0.001) when the SDI remained below 0.67.

## Discussion

Our study revealed that the age-standardized incidence rate of diabetes increases from 249 to 329 per 100,000 during 1990-2019, which was substantially lower than the global average. The estimated annual percentage change of the incidence in China (1.02%) was also lower than the global rate (1.57%), consistent with previous findings (1). Since 2005, the incidence of type 2 diabetes (T2DM) has been reported to decline in some developed countries, such as Sweden (11). Our study indicated that the aged-standardized prevalence rate of diabetes in China grew from 4788 to 8170 per 100,000 between 1990 and 2019, accounting for one-fifth of the global burden and the largest population of diabetic patients. The prevalence of diabetes in other countries also exhibited an upward trend, such as the United States (12). Our research showed that age-standardized mortality rate of diabetes in China rose from 9 per 100,000 to 15 per 100,000 , lower than the global rate. The proportion of total global diabetes deaths also increased over the same period (from 10.6% to 11.2%).

The increasing burden of diabetes in China is likely attributed to the heightened exposure to risk factors. T2DM constitutes the most prevalent form of diabetes, comprising over 90% of all cases worldwide (13). T2DM is caused by multiple factors, including metabolic factors (such as overweight and obesity), environmental factors, and behavioral factors (such as tobacco exposure, dietary factors, and low physical activity) (14). Overweight and obesity serve as significant risk factors for T2DM (15). According to the China Health and Nutrition Survey, the prevalence of overweight and obesity among individuals aged 18 years and above increased from 8.0% and 2.9% to 17.1% and 11.4% for males, and from 10.7% and 5.0% to 14.4% and 10.1% for females during 1993–2009, respectively (16).

Research indicates that economic development has resulted in significant environmental pollution, which may negatively impact glucose metabolism, resulting in insulin resistance and T2DM (17). Meta-analyses have demonstrated a positive correlation between major atmospheric pollutants (such as PM_2.5_, PM_10_, NO_2_, and NO_X_) and T2DM (18). PM_2.5_ levels have increased from 31.2 μg/m^3^ in 2000 to 97.0 μg/m^3^ in 2015 across 15 provinces and regions in China (19). A separate meta-analyze has shown a 10% increased risk of T2DM per 10-μg/m^3^ increase in PM_2.5_ exposure (20). According to the 2015 China Adult Tobacco Survey Report, the current prevalence of smoking among individuals aged 15 and above is 27.7%, with approximately 316 million smokers in China (21). Smoking may contribute to T2DM by promoting fat accumulation, thereby leading to insulin resistance (22). Some countries have implemented effective smoking cessation policies to control population-level smoking exposure, such as Australia (23).

Low dietary intake of whole grains is significantly associated with an increased risk of T2DM (24). According to the Dietary Guidelines for Chinese Residents 2016 (25), whole grain consumption in China is low, possibly due to high intake of refined grains. Li et al. (26) suggested that a diet high in refined grains and low in whole grain is a primary factor contributing to the increasing burden of diabetes in China. Furthermore, the risk of T2DM increases as fruit intake decreases, according to a linear dose-response meta-analysis (27). Long-term consumption of red and processed meat can reduce insulin sensitivity, elevating the risk of T2DM (28). However, as global meat prices have declined, red and processed meat has become more accessible in low- and middle-income countries (29). Health-motivated taxes on red and processed meat are low in these countries, including China (30), potentially increasing processed meat consumption and the burden of T2DM. Concurrently, China’s economic development has shifted work models from manual labor to mental labor, contributing to decreased physical activity (31). Engaging in low, moderate, and high-intensity activities can reduce the risk of developing T2DM by 25%–40% (32). This study further found that the incidence, prevalence, and mortality of diabetes are influenced by the level of social development or macroeconomic factors. This is consistent with other research findings (14). Additionally, diabetes heightens the risk of complications. Recent research has demonstrated the efficacy of novel anti-diabetic medications in lowering the incidence of diabetic complications. This discovery not only presents more effective therapeutic choices for individuals with diabetes but also holds promise in mitigating the long-term health impacts of the disease’s complications (33).

To assess the impact of population growth, aging, and epidemiologic changes on the epidemiology of diabetes over the past three decades, we conducted a decomposition analysis of raw DALYs by population, age structure, and age and population standardized morbidity and mortality rates (which we are referring to here as epidemiologic changes). The global overall increase in burden of diabetes is largely attributable to population growth and epidemiologic changes, whereas in China, it is primarily driven by population growth and aging. From a global perspective, the increasing burden of diabetes is influenced by two main factors. Firstly, the expansion of the global population has resulted in a higher number of individuals affected by diabetes. Secondly, epidemiologic changes, reflecting shifts in lifestyles and environmental pollution worldwide, are also contributing to the mounting burden of diabetes (34). The fast-paced modern lifestyle, characterized by high-sugar and high-fat dietary habits and sedentary behaviors, is fueling a global surge in overweight and obesity prevalence, thereby increasing the risk of diabetes (35). In China, the burden of diabetes is chiefly shaped by population growth and aging. Aging has intensified the onset of chronic diseases, with diabetes disproportionately affecting the elderly, thereby amplifying China’s diabetes DALYs. Moreover, rapid economic development and urbanization in China have brought about significant changes in lifestyles and dietary structures (36), leading to increasing risk of diabetes in the country (37). To effectively address the increasing burden of diabetes, orchestrated health intervention strategies need to be implemented globally. In particular, targeted measures for diabetes prevention and control are imperative to confront the challenges posed by population aging and lifestyle changes in China.

This study has several limitations. Firstly, as most of the data were derived from GBD 2019, similar limitations in estimates of incidence, prevalence, deaths, and DALYs burden in the GBD study also apply to this study. Secondly, our study did not analyze population characteristics of the diabetes disease burden in China; instead, it focused on temporal trend analysis. Lastly, while many previous studies focused on adults, our investigation included individuals aged 20 years or older.

## Conclusion

The diabetes DALYs among adults aged 20 years or older has grown in China and worldwide, but the proportion of new cases and patients has decreased. The overall progression of the diabetes DALYs in China is more pronounced compared to other countries. The global rise in diabetes DALYs is largely attributed to population growth and epidemiological changes, whereas the burden mainly driven by population growth and aging in China.

## Data availability statement

The original contributions presented in the study are included in the article/**Supplementary Material**. Further inquiries can be directed to the corresponding author.

## Author contributions

LJ: Writing – original draft, Formal analysis, Data curation. MS: Writing – review & editing, Supervision, Software. GZ: Supervision, Writing – review & editing. LZ: Supervision, Methodology, Investigation, Writing – review & editing.
