# Supplementary material for: Investigating the temporal trends of diabetes disease burden in China during 1990-2019 from a global perspective
Source: Front Endocrinol (Lausanne). 2024 May 10;15:1324318. doi: 10.3389/fendo.2024.1324318 (PMC11116686; doi:10.3389/fendo.2024.1324318)
Supplement: Supplementary file 1 [file DataSheet_1.pdf]

Supplementary appendix

Supplement to: Investigating the temporal trends of diabetes disease burden in China during 1990-2019 from a global perspective

Table of Content

1. Definition of diabetes and ICD code……2

2. Decomposition analysis……2

Figure S1. The temporal trends of ASIR, ASPR, and ASMR of diabetes in China and globally during 1990–2019……3

Figure S2. ASIR of diabetes in 1990 (a), 2019 (b) in 203 countries and territories……4

Figure S3. ASPR of diabetes in 1990 (a) and 2019 (b) in 203 countries and territories……5

Figure S4. Number of people with diabetes in 1990 (a) and 2019 (b) in 203 countries and territories……6

Figure S5. ASMR of diabetes in 1990 (a), 2019 (b) in 203 countries and territories……7

Table S1. The temporal trends and ranking of incidence and mortality of diabetes during 1990–2019……8

Table S2. Changes in DALYs number of diabetes according to population-level determinants of aging, population growth, and epidemiological change from 1990 to 2019 in China and globally ……13

1. Definition of diabetes and ICD code

For the definition and classification of diabetes, we refer to the criteria given in the GBD studies[1]. The definitions and diagnostic criteria for diabetes mellitus are presented in table 1 below. In our study, diabetes (ICD: E10-E10.1, E10.3-E11.1, E11.3-E11.9, P70.2) included only type 1 diabetes mellitus (ICD: E10-E10.1, E10.3-E10.9, P70.2), and type 2 diabetes mellitus (ICD: E11-E11.1, E11.3-E11.9).

Table 1. Overall Diabetes Mellitus

Criterion	Definition
Diabetes Mellitus parent	Fasting plasma glucose (FPG) ≥ 126 mg/dL (7 mmol/L), or reporting to be on treatment with drugs or insulin for diabetes, or persons <15 years who are diagnosed by physicians and identified through a diabetic registry or hospital records.

2. Decomposition analysis

We applied the decomposition methodology introduced by Das Gupta[2] to disentangle diabetes DALYs by considering population age structure, population growth, and epidemiological alterations. The calculation of DALYs for each specific location was accomplished employing the subsequent formula:

$$\text{DALY}_{ay,\ py,\ ey} = \sum_{i=1}^{16}(a_{i,y} * p_y * e_{i,y})$$

Herein, $\text{DALY}_{ay,\ py,\ ey}$ delineates the DALYs attributed to the combined factors of age structure, population, and DALYs rate for a specific year denoted as y; where $a_{i,y}$ signifies the proportion of the population falling within age category i of the 16 age categories in the given year y; p_y denotes the total population for the specific year y; and $e_{i,y}$ signifies the DALYs rate pertaining to age category i during the year y. The quantified impact of each individual factor on the change in DALYs between the years 1990 and 2019 was discerned through the variation of one factor, while keeping all other variables constant. For instance, the influence of age structure was calculated as follows:

$[(\text{DALY}_{a2019,\ p1990,\ e1990} + \text{DALY}_{a2019,\ p2019,\ e2019})/3 + (\text{DALY}_{a2019,\ p1990,\ e2019} + \text{DALY}_{a2019,\ p2019,\ e1990})/6] - [(\text{DALY}_{a1990,\ p2019,\ e2019} + \text{DALY}_{a1990,\ p1990,\ e1990})/3 + (\text{DALY}_{a1990,\ p2019b,\ e1990} + \text{DALY}_{a1990,\ p1990,\ e2019})/6]$

References:

1. GBD 2019 Diseases and Injuries Collaborators. Global burden of 369 diseases and injuries in 204 countries and territories, 1990-2019: a systematic analysis for the Global Burden of Disease Study 2019. Lancet 2020; 396(10258): 1204-22.
2. Chevan A, Sutherland M. Revisiting Das Gupta: refinement and extension of standardization and decomposition. Demography 2009; 46(3): 429-49.

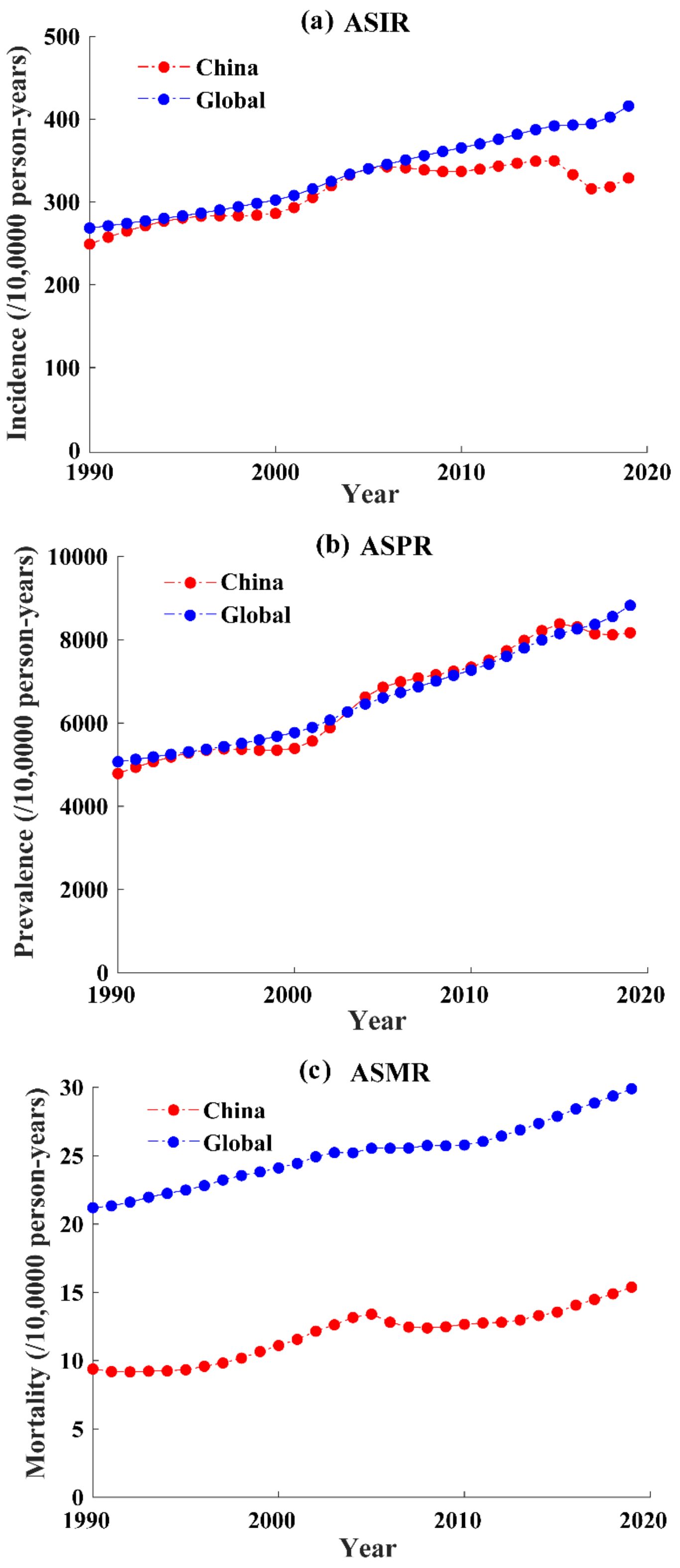

Figure S1. The temporal trends of ASIR, ASPR, and ASMR of diabetes in China and globally during 1990–2019

(ASIR: age-standardized incidence rate; ASPR: age-standardized prevalence rate; ASMR: age-standardized mortality rate)

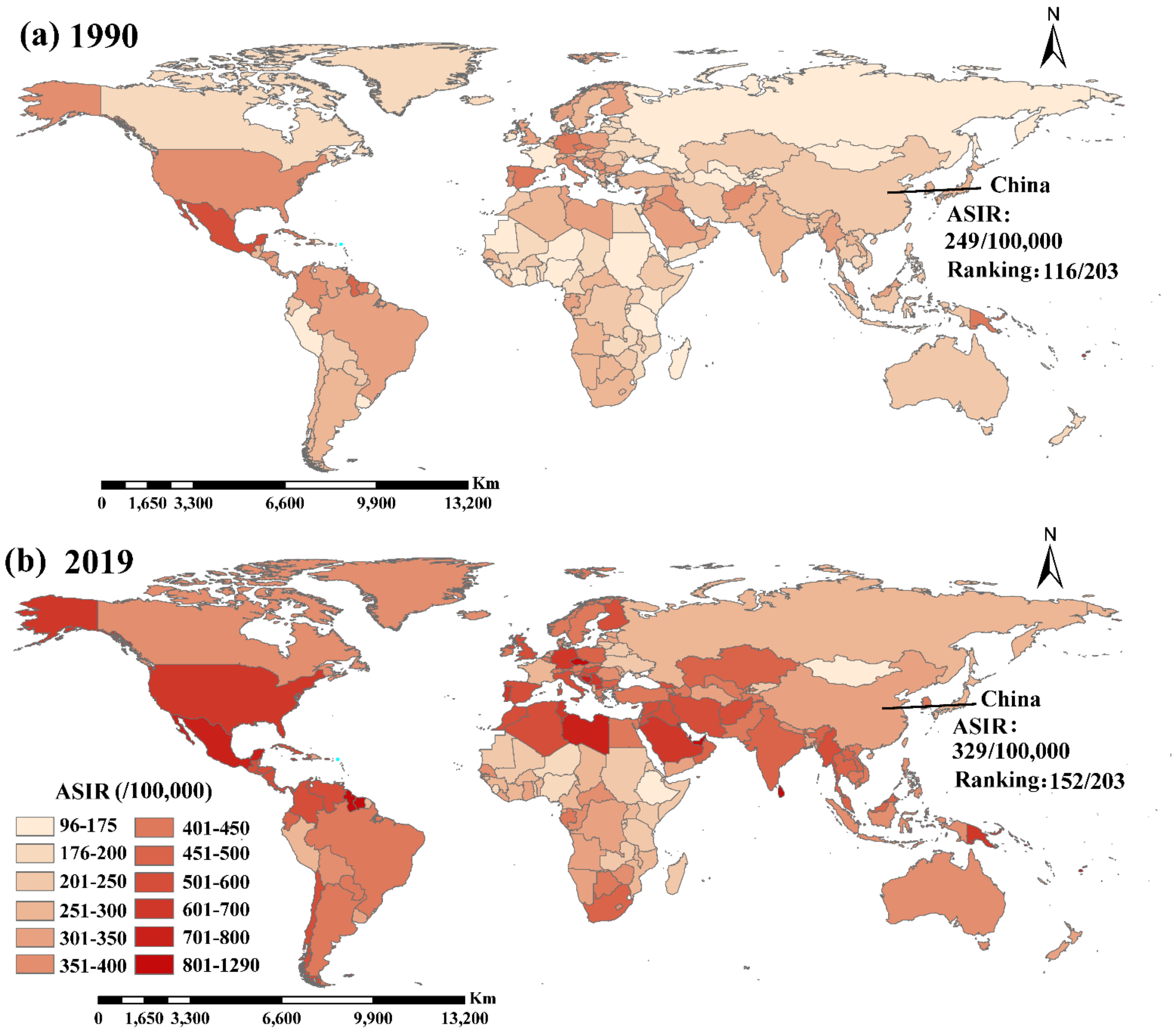

Figure S2. ASIR of diabetes in 1990 (a), 2019 (b) in 203 countries and territories (ASIR: age-standardized incidence)

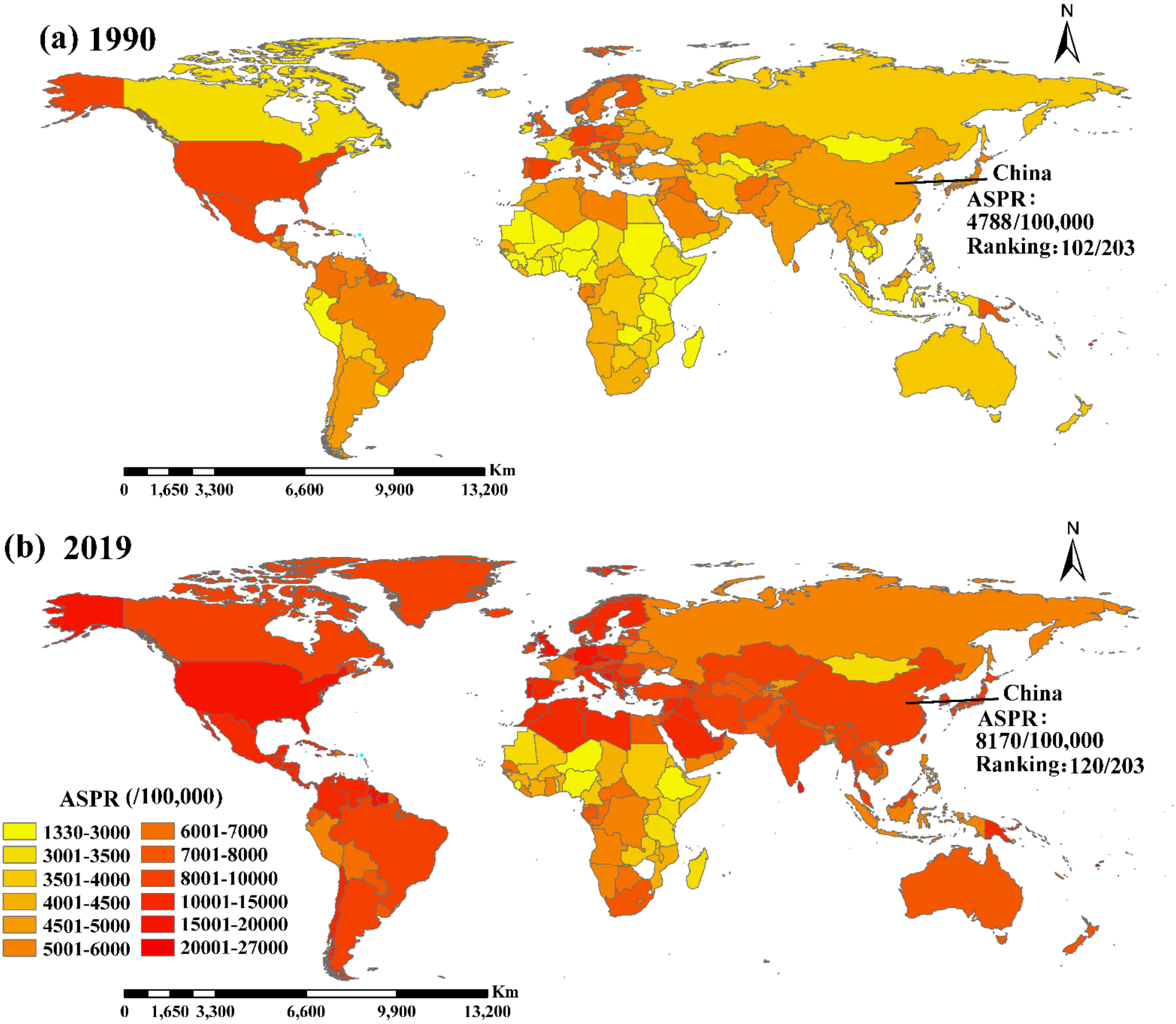

Figure S3. ASPR of diabetes in 1990 (a) and 2019 (b) in 203 countries and territories (ASPR: age-standardized prevalence rate)

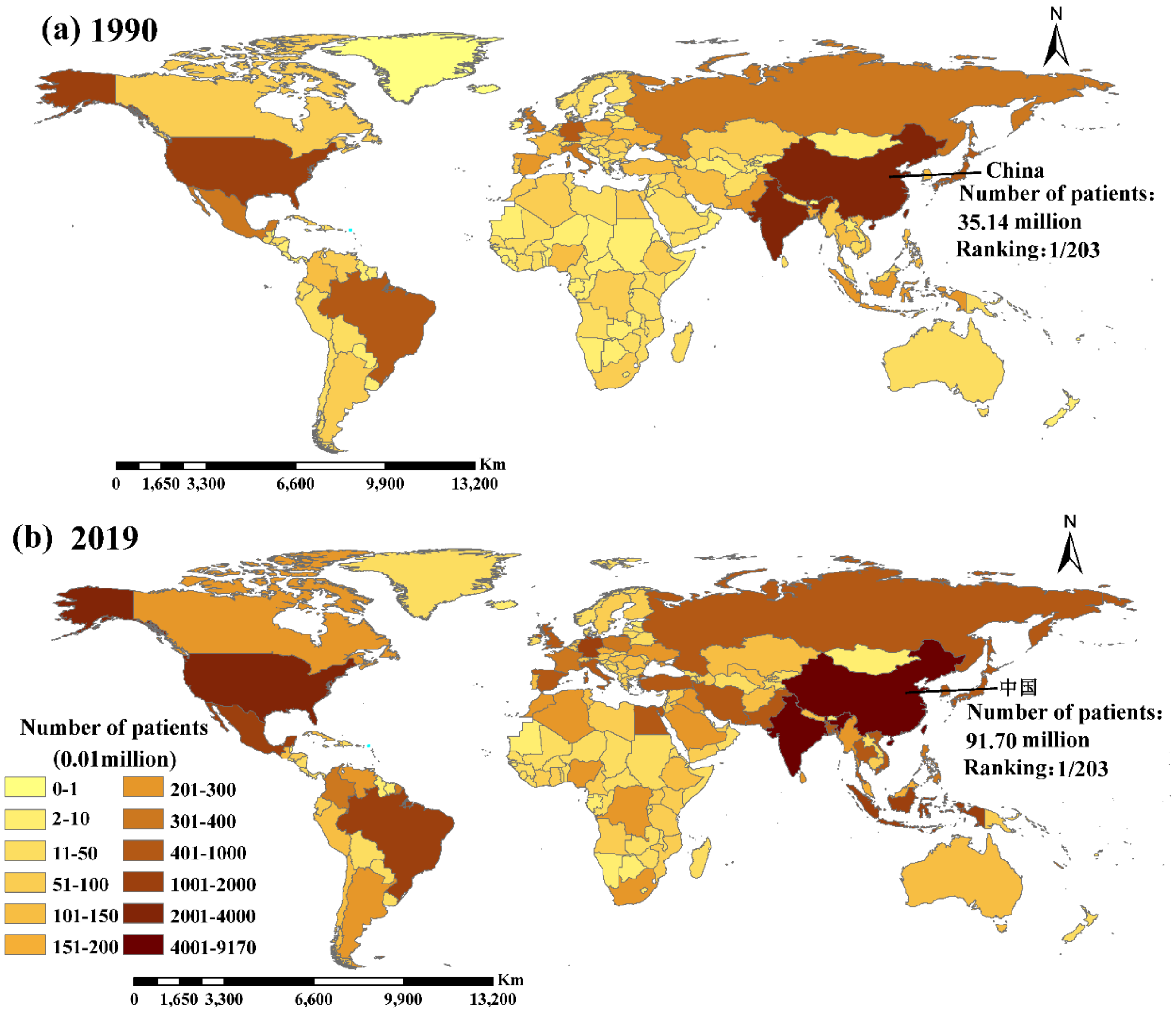

Figure S4. Number of people with diabetes in 1990 (a) and 2019 (b) in 203 countries and territories

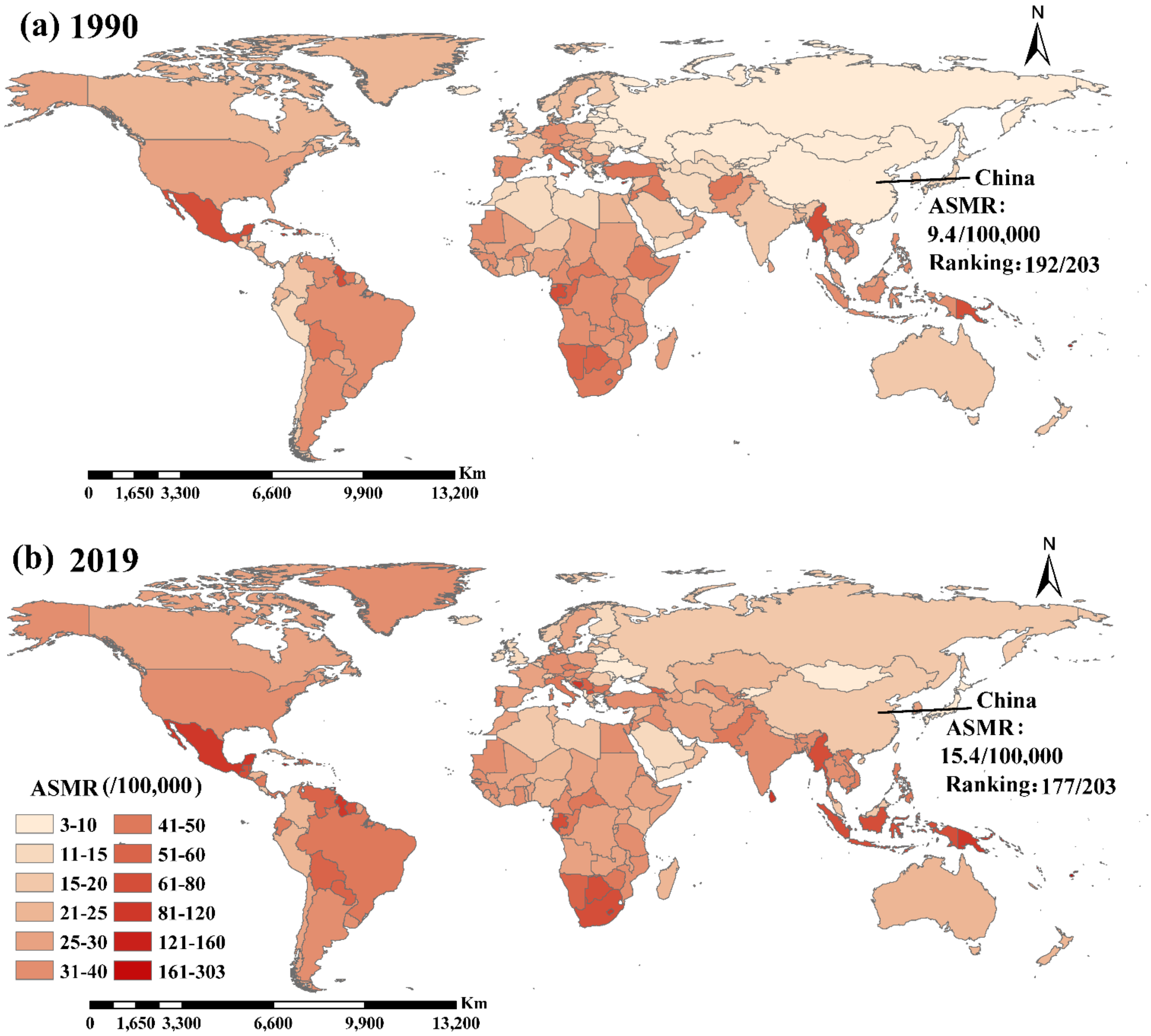

Figure S5. ASMR of diabetes in 1990 (a), 2019 (b) in 203 countries and territories (ASMR: age-standardized mortality rate)

Table S1. The temporal trends and ranking of incidence and mortality of diabetes during 1990–2019 (EAPCs: Estimated annual percentage change,%)

location		Age-standardized incidence rate			Age-standardized mortality rate	
		Ranking	EAPCs(%)		Ranking	EAPCs(%)
Global		-	1.57(1.53–1.65)		-	1.07(1.01–1.15)
Afghanistan		67	2.00(1.81–2.22)		153	-0.5(-0.82–-0.18)
Albania		55	2.10(2.00–2.24)		66	1.26(0.77–1.76)
Algeria		25	2.68(2.58–2.84)		64	1.27(0.92–1.64)
American Samoa		80	1.88(1.63–2.17)		20	2.56(2.3–2.89)
Andorra		34	2.50(2.46–2.61)		46	1.65(1.52–1.82)
Angola		173	1.01(0.91–1.12)		143	-0.36(-0.50–-0.22)
Antigua and Barbuda		88	1.79(1.75–1.87)		157	-0.59(-0.87–-0.31)
Argentina		159	1.15(1.02–1.31)		160	-0.65(-0.95–-0.33)
Armenia		52	2.13(1.93–2.38)		42	1.75(0.88–2.66)
Australia		92	1.77(1.50–2.08)		109	0.41(0.09–0.73)
Austria		38	2.39(2.29–2.55)		70	1.15(0.71–1.60)
Azerbaijan		15	2.98(2.91–3.13)		52	1.55(1.07–2.06)
Bahamas		98	1.74(1.73–1.78)		134	-0.16(-0.39–0.07)
Bahrain		6	3.53(3.45–3.73)		54	1.47(1.13–1.85)
Bangladesh		102	1.68(1.58–1.81)		82	0.94(0.63–1.26)
Barbados		143	1.37(1.35–1.41)		146	-0.43(-0.64–-0.22)
Belarus		193	0.53(0.41–0.66)		202	-3.45(-3.99–-2.79)
Belgium		133	1.47(1.46–1.50)		175	-1.05(-1.20–-0.9)
Belize		68	1.99(1.90–2.12)		89	0.82(0.37–1.28)
Benin		136	1.42(1.37–1.50)		117	0.16(-0.01–0.32)
Bermuda		105	1.66(1.63–1.72)		129	-0.11(-0.34–0.12)
Bhutan		69	1.99(1.96–2.06)		23	2.52(2.46–2.64)
Bolivia		131	1.49(1.44–1.56)		81	0.94(0.89–1.00)
Bosnia and Herzegovina		10	3.17(3.07–3.38)		2	5.78(5.40–6.50)
Botswana		76	1.92(1.83–2.05)		92	0.76(0.39–1.14)
Brazil		157	1.16(1.04–1.30)		87	0.84(0.77–0.92)
Brunei Darussalam		86	1.83(1.58–2.10)		127	-0.05(-0.31–0.22)
Bulgaria		155	1.18(1.07–1.30)		123	-0.01(-0.39–0.37)
Burkina Faso		188	0.69(0.65–0.73)		166	-0.83(-0.99–-0.67)
Burundi		199	0.16(0.08–0.24)		193	-2.17(-2.38–-1.91)
Cabo Verde		74	1.96(1.93–2.04)		24	2.50(1.81–3.27)
Cambodia		20	2.88(2.70–3.15)		114	0.32(0.18–0.45)
Cameroon		147	1.32(1.11–1.55)		103	0.5(0.23–0.77)
Canada		60	2.04(2.00–2.12)		140	-0.34(-0.97–0.29)
Central African Republic		145	1.33(1.30–1.37)		136	-0.23(-0.27–-0.18)
Chad		170	1.04(0.93–1.16)		122	0.01(-0.26–0.27)
Chile		30	2.55(2.40–2.77)		63	1.28(1.15–1.43)
China		171	1.02(0.79–1.26)		41	1.75(1.54–2.00)

Colombia		178	0.94(0.8–1.1)		149	-0.45(-0.85–-0.04)
Comoros		187	0.71(0.68–0.76)		128	-0.09(-0.22–0.05)
Congo		164	1.1(1.02–1.2)		174	-1.02(-1.23–-0.81)
Cook Islands		66	2(1.83–2.21)		56	1.43(1.33–1.55)
Costa Rica		123	1.55(1.49–1.63)		156	-0.58(-1.16–0.01)
Cote d'Ivoire		111	1.63(1.57–1.72)		105	0.47(0.16–0.79)
Croatia		148	1.26(1.2–1.32)		85	0.87(0.6–1.15)
Cuba		138	1.4(1.24–1.59)		180	-1.4(-2.21–-0.56)
Cyprus		201	-0.12(-0.3–0.06)		200	-2.76(-2.97–-2.47)
Czechia		62	2.03(1.84–2.26)		9	3.37(2.34–4.51)
Democratic People's Republic of Korea		108	1.64(1.62–1.68)		86	0.85(0.74–0.96)
Democratic Republic of the Congo		172	1.02(0.96–1.08)		170	-0.94(-1.01–-0.87)
Denmark		31	2.53(2.42–2.7)		65	1.27(0.79–1.76)
Djibouti		134	1.47(1.43–1.53)		62	1.31(1.21–1.41)
Dominica		90	1.78(1.73–1.87)		152	-0.5(-0.7–-0.29)
Dominican Republic		27	2.59(2.5–2.75)		17	2.69(2.52–2.93)
Ecuador		35	2.48(2.28–2.73)		28	2.34(2.06–2.68)
Egypt		14	2.98(2.88–3.17)		53	1.48(1.36–1.62)
El Salvador		37	2.42(2.32–2.59)		8	3.44(3.18–3.83)
Equatorial Guinea		192	0.63(0.56–0.7)		168	-0.87(-1.08–-0.65)
Eritrea		166	1.07(1.05–1.1)		111	0.37(0.26–0.48)
Estonia		139	1.4(1.14–1.68)		61	1.35(0.51–2.23)
Eswatini		97	1.75(1.53–2)		32	2.24(1.64–2.9)
Ethiopia		203	-1.08(-1.2–-0.95)		197	-2.51(-2.67–-2.3)
Fiji		57	2.07(1.88–2.31)		21	2.54(2.25–2.89)
Finland		117	1.6(1.48–1.74)		184	-1.44(-1.72–-1.14)
France		83	1.86(1.75–2)		72	1.15(0.66–1.65)
Gabon		153	1.2(1.15–1.27)		145	-0.43(-0.55–-0.3)
Gambia		89	1.79(1.7–1.9)		37	1.8(1.51–2.13)
Georgia		12	3.05(2.95–3.24)		5	4.16(3.88–4.61)
Germany		149	1.24(1.08–1.41)		158	-0.59(-0.88–-0.3)
Ghana		53	2.12(1.85–2.43)		45	1.68(1.27–2.12)
Greece		126	1.52(1.47–1.6)		75	1.03(0.49–1.58)
Greenland		7	3.42(3.42–3.53)		133	-0.14(-0.36–0.07)
Grenada		64	2.01(1.93–2.13)		151	-0.48(-0.79–-0.17)
Guam		112	1.63(1.61–1.67)		80	0.94(0.68–1.22)
Guatemala		40	2.33(2.08–2.63)		6	4(3.39–4.78)
Guinea		169	1.05(0.88–1.24)		110	0.38(-0.01–0.78)
Guinea-Bissau		177	0.97(0.94–1.01)		137	-0.29(-0.45–-0.12)
Guyana		72	1.97(1.79–2.19)		60	1.36(0.98–1.76)
Haiti		154	1.18(1.14–1.23)		176	-1.15(-1.25–-1.03)
Honduras		144	1.35(1.33–1.39)		47	1.65(1.5–1.82)

Hungary		129	1.5(1.28–1.74)		71	1.15(0.85–1.46)
Iceland		33	2.5(2.52–2.56)		131	-0.12(-0.28–0.04)
India		107	1.64(1.57–1.74)		35	1.94(1.83–2.1)
Indonesia		82	1.87(1.67–2.11)		27	2.37(2.21–2.59)
Iran		22	2.8(2.73–2.95)		10	3.26(2.98–3.65)
Iraq		118	1.59(1.55–1.66)		164	-0.79(-0.97–-0.61)
Ireland		4	3.67(3.54–3.94)		186	-1.54(-1.72–-1.34)
Israel		61	2.03(1.65–2.45)		107	0.42(-0.52–1.38)
Italy		114	1.61(1.11–2.14)		118	0.13(0.02–0.23)
Jamaica		73	1.97(1.87–2.1)		88	0.82(0.55–1.1)
Japan		202	-0.16(-0.34–0.02)		173	-1(-1.51–-0.48)
Jordan		142	1.38(1.29–1.48)		187	-1.61(-2.06–-1.12)
Kazakhstan		36	2.42(2.35–2.56)		79	0.96(0.12–1.82)
Kenya		168	1.06(1.05–1.07)		106	0.47(0.34–0.6)
Kiribati		96	1.75(1.58–1.96)		93	0.76(0.37–1.17)
Kuwait		109	1.63(1.42–1.87)		182	-1.42(-2.37–-0.43)
Kyrgyzstan		127	1.52(1.5–1.56)		189	-1.63(-2.13–-1.1)
Lao People's Democratic Republic		125	1.53(1.48–1.6)		165	-0.81(-0.91–-0.7)
Latvia		79	1.88(1.74–2.06)		15	2.73(2.34–3.2)
Lebanon		106	1.65(1.6–1.73)		119	0.09(-0.03–0.22)
Lesotho		101	1.71(1.63–1.81)		12	2.84(2.49–3.27)
Liberia		184	0.78(0.58–0.99)		178	-1.34(-1.5–-1.16)
Libya		24	2.69(2.58–2.88)		69	1.17(0.85–1.51)
Lithuania		163	1.12(0.97–1.28)		91	0.76(0.5–1.03)
Luxembourg		1	4.57(4.53–4.83)		179	-1.35(-1.46–-1.23)
Madagascar		183	0.8(0.73–0.87)		172	-0.98(-1.08–-0.87)
Malawi		186	0.74(0.64–0.84)		161	-0.71(-0.97–-0.44)
Malaysia		103	1.68(1.57–1.81)		195	-2.34(-2.78–-1.84)
Maldives		189	0.69(0.57–0.81)		201	-2.89(-3.11–-2.59)
Mali		175	1(0.99–1.03)		135	-0.16(-0.3–-0.02)
Malta		132	1.48(1.38–1.6)		139	-0.33(-0.57–-0.09)
Marshall Islands		63	2.02(1.88–2.2)		51	1.56(1.5–1.65)
Mauritania		185	0.77(0.64–0.9)		138	-0.3(-0.35–-0.26)
Mauritius		3	3.81(3.58–4.19)		1	6.45(5.68–7.65)
Mexico		162	1.14(0.99–1.3)		73	1.11(0.87–1.36)
Micronesia		23	2.75(2.41–3.17)		29	2.3(2.11–2.55)
Monaco		47	2.24(2.24–2.29)		102	0.51(0.32–0.7)
Mongolia		48	2.23(2.11–2.41)		126	-0.05(-0.22–0.13)
Montenegro		100	1.71(1.67–1.78)		39	1.78(1.57–2.03)
Morocco		16	2.96(2.95–3.06)		16	2.7(2.59–2.88)
Mozambique		165	1.1(0.98–1.23)		99	0.65(0.39–0.91)
Myanmar		119	1.59(1.49–1.72)		121	0.03(-0.05–0.11)
Namibia		194	0.52(0.47–0.57)		148	-0.44(-0.83–-0.05)

Nauru		58	2.07(2.06–2.12)		90	0.78(0.54–1.02)
Nepal		71	1.97(1.87–2.11)		18	2.62(2.31–3.01)
Netherlands		167	1.06(1.03–1.1)		183	-1.42(-1.78–-1.04)
New Zealand		130	1.49(1.26–1.74)		159	-0.62(-1.07–-0.16)
Nicaragua		110	1.63(1.57–1.72)		44	1.71(1.39–2.05)
Niger		54	2.1(1.85–2.4)		98	0.67(0.62–0.73)
Nigeria		198	0.2(0.03–0.38)		155	-0.57(-0.61–-0.53)
Niue		91	1.78(1.63–1.96)		59	1.38(1.04–1.74)
North Macedonia		41	2.33(2.18–2.53)		11	2.86(2.47–3.34)
Northern Mariana Islands		13	3.03(2.89–3.25)		3	5.09(4.57–5.87)
Norway		182	0.82(0.69–0.94)		141	-0.35(-0.78–0.07)
Oman		160	1.15(0.87–1.45)		171	-0.98(-1.61–-0.33)
Pakistan		78	1.91(1.83–2.02)		38	1.8(1.65–1.97)
Palau		28	2.56(2.45–2.74)		14	2.82(2.78–2.94)
Palestine		46	2.25(2.11–2.45)		130	-0.11(-0.62–0.39)
Panama		56	2.08(2.05–2.15)		25	2.43(2.2–2.71)
Papua New Guinea		115	1.61(1.58–1.65)		96	0.7(0.58–0.83)
Paraguay		95	1.75(1.61–1.93)		13	2.83(2.41–3.32)
Peru		65	2.01(1.93–2.13)		48	1.63(1.35–1.95)
Philippines		158	1.16(1.09–1.24)		77	1.01(0.82–1.22)
Poland		141	1.38(1.14–1.65)		100	0.6(0.12–1.09)
Portugal		94	1.76(1.61–1.95)		116	0.18(-0.06–0.41)
Puerto Rico		146	1.32(1.13–1.53)		67	1.24(1–1.49)
Qatar		26	2.6(2.01–3.26)		199	-2.6(-3.53–-1.59)
Republic of Korea		116	1.6(1.5–1.73)		84	0.9(0.14–1.67)
Republic of Moldova		161	1.14(0.98–1.31)		188	-1.61(-2.06–-1.14)
Romania		140	1.39(1.31–1.49)		97	0.7(0.32–1.08)
Russian Federation		137	1.41(1.32–1.53)		36	1.84(1.02–2.71)
Rwanda		200	0.16(-0.01–0.33)		198	-2.53(-3.03–-1.96)
Saint Kitts and Nevis		151	1.22(1.06–1.39)		191	-1.83(-2.13–-1.49)
Saint Lucia		135	1.44(1.4–1.5)		169	-0.89(-1.28–-0.5)
Saint Vincent and the Grenadines		104	1.67(1.64–1.73)		115	0.29(0.03–0.55)
Samoa		84	1.86(1.71–2.04)		68	1.2(0.92–1.5)
San Marino		70	1.98(1.93–2.06)		76	1.02(0.91–1.14)
Sao Tome and Principe		179	0.93(0.88–0.99)		150	-0.46(-0.64–-0.27)
Saudi Arabia		59	2.05(1.99–2.15)		196	-2.44(-2.88–-1.95)
Senegal		120	1.58(1.4–1.79)		74	1.04(0.87–1.21)
Serbia		128	1.5(1.44–1.59)		40	1.77(1.52–2.04)
Seychelles		8	3.27(3.25–3.4)		58	1.4(1.28–1.55)
Sierra Leone		152	1.21(1.17–1.26)		132	-0.14(-0.33–0.05)
Singapore		197	0.37(0.2–0.53)		203	-5.96(-6.87–-4.68)
Slovakia		156	1.17(1.1–1.24)		163	-0.75(-0.98–-0.51)
Slovenia		196	0.49(0.34–0.64)		192	-1.9(-2.75–-1)

Solomon Islands		43	2.32(2.31–2.39)		43	1.71(1.55–1.9)
Somalia		191	0.63(0.58–0.69)		144	-0.38(-0.53–-0.23)
South Africa		81	1.88(1.78–2.01)		33	2.23(1.8–2.71)
South Sudan		174	1(0.84–1.18)		147	-0.43(-0.75–-0.11)
Spain		190	0.65(0.54–0.76)		177	-1.26(-1.43–-1.08)
Sri Lanka		5	3.61(3.59–3.76)		4	5.06(4.73–5.65)
Sudan		39	2.35(2.32–2.43)		101	0.53(0.39–0.68)
Suriname		32	2.51(2.41–2.68)		55	1.47(1.06–1.89)
Sweden		122	1.56(1.39–1.75)		104	0.48(0.21–0.75)
Switzerland		150	1.23(1.12–1.35)		194	-2.19(-2.49–-1.83)
Syrian Arab Republic		42	2.33(2.06–2.65)		142	-0.36(-1.21–0.5)
Tajikistan		9	3.25(3.16–3.45)		26	2.41(2.04–2.85)
Thailand		87	1.81(1.6–2.04)		112	0.33(-0.09–0.76)
Timor-Leste		19	2.89(2.85–3.01)		31	2.26(2.13–2.46)
Togo		121	1.58(1.54–1.64)		83	0.93(0.83–1.05)
Tokelau		85	1.84(1.79–1.92)		95	0.72(0.4–1.05)
Tonga		124	1.53(1.51–1.57)		57	1.42(1.25–1.6)
Trinidad and Tobago		176	0.99(0.93–1.06)		120	0.03(-0.26–0.33)
Tunisia		21	2.86(2.8–3.01)		30	2.28(2.22–2.39)
Turkey		17	2.95(2.42–3.57)		154	-0.55(-0.85–-0.25)
Turkmenistan		29	2.56(2.52–2.65)		50	1.58(1.21–1.99)
Tuvalu		75	1.95(1.84–2.1)		78	0.96(0.68–1.26)
Uganda		181	0.91(0.87–0.96)		162	-0.72(-0.9–-0.54)
Ukraine		180	0.91(0.79–1.05)		185	-1.48(-1.95–-0.98)
United Arab Emirates		45	2.26(1.88–2.69)		181	-1.41(-2.19–-0.61)
United Kingdom		44	2.27(2.15–2.44)		190	-1.69(-1.86–-1.48)
United Republic of Tanzania		93	1.76(1.67–1.89)		124	-0.01(-0.07–0.05)
United States of America		11	3.1(2.67–3.63)		125	-0.04(-0.48–0.4)
United States Virgin Islands		50	2.16(2.09–2.27)		22	2.53(2.35–2.77)
Uruguay		18	2.89(2.48–3.39)		113	0.32(0.19–0.46)
Uzbekistan		2	3.99(3.89–4.26)		7	3.57(3.07–4.21)
Vanuatu		51	2.14(2.14–2.2)		19	2.62(2.5–2.81)
Venezuela		77	1.91(1.79–2.06)		49	1.63(1.32–1.96)
Viet Nam		49	2.16(1.98–2.4)		94	0.75(0.47–1.05)
Yemen		113	1.62(1.52–1.76)		108	0.41(0.25–0.58)
Zambia		195	0.5(0.46–0.54)		167	-0.84(-1.11–-0.56)
Zimbabwe		99	1.72(1.7–1.77)		34	2.11(1.78–2.48)

Table S2. Changes in DALYs number of diabetes according to population-level determinants of aging, population growth, and epidemiological change from 1990 to 2019 in China and globally

**Location**	**Overall difference [a]**	**Change due to Population-level determinants (% contribute to the total changes)**		
		**Population growth [b]**	**Epidemiologic changes [c]**	**Aging [d]**
**China**	5,720,690	2,798,602 (48.9%)	723,773 (7.4%)	2,498,315 (43.7%)
**Global**	42,296,649	23,326,814 (55.2%)	10,397,797 (24.6%)	8,572,039 (20.3%)

a. Change in DALYs number during 1990-2019;
b. Change in DALYs number due to change in population number;
c. Change in DALYs number due to change in epidemiologic changes. Epidemiologic changes refer to the DALYs number change when age structure and population hold constant;
d. Change in DALYs number due to the age structure.
